# The Effect of Sex on the Remimazolam Dosage Required for Successful i-gel Supraglottic Airway Insertion with Remifentanil in Non-Paralyzed Patients: An Up-and-Down Sequential Allocation Trial

**DOI:** 10.3390/jcm13030670

**Published:** 2024-01-24

**Authors:** Ju-Yeon Oh, Sung-Yong Park, Jung-Yoon Moon, Ji-Hyun Park, Han-Bum Joe

**Affiliations:** 1 Department of Anesthesiology and Pain Medicine, Ajou University School of Medicine, Suwon 16499, Republic of Korea; itisamy@hanmail.net (J.-Y.O.); anepark@hanmail.net (S.-Y.P.); jymoon1028@gmail.com (J.-Y.M.); 2 Office of Biostatistics, Medical Research Collaborating Center, Ajou Research Institute for Innovative Medicine, Ajou University Medical Center, Suwon 16499, Republic of Korea; jhn1105@gmail.com

**Keywords:** airway management, general anesthesia, remifentanil, remimazolam, sex, supraglottic airway

## Abstract

**(1) Background:** The physiological and pharmacological variations between men and women are known to influence drug efficacy. The objective of this study was to determine the 50% and 95% effective doses (ED_50_ and ED_95_) of remimazolam required for i-gel supraglottic airway (ISA) insertion under remifentanil infusion without neuromuscular blocking agents (NMBAs) in both males and females. **(2) Methods:** Patients aged 19–65 years, scheduled for general anesthesia using ISA, were enrolled in this study. Patients were divided into two groups based on their sex. The anesthesia process began with a remifentanil infusion targeting an effect-site concentration of 3.0 ng/mL, accompanied by a remimazolam injection. The initial remimazolam dose was 0.25 mg/kg, and it was adjusted with a step size of 0.05 mg/kg based on the outcome of ISA insertion in the preceding patient. **(3) Results:** The ED_50_ of remimazolam (mean ± standard error) was 0.28 ± 0.02 mg/kg in the male group and 0.18 ± 0.02 mg/kg in the female group (*p* < 0.001). Additionally, ED_95_, which was calculated using the isotonic regression method, was significantly comparable between the male and female groups (male: 0.35 mg/kg, 95% confidence interval [CI] = 0.34–0.35; female: 0.29 mg/kg, 95% CI = 0.25–0.30). **(4) Conclusions:** This study showed that both the ED_50_ and the ED_95_ of remimazolam for successful ISA insertion was higher for men than that for women. Therefore, while using remimazolam alongside remifentanil infusion without NMBAs for ISA insertion, one should consider the patient’s sex for appropriate dosing.

## 1. Introduction

Remimazolam (CNS 7056), a commonly used, short-acting benzodiazepine for procedural and deep sedation, offers several advantages [1,2]. A meta-analysis revealed that remimazolam posed a lower risk of post-induction hypotension and injection pain than propofol [3]. In addition to the hemodynamic stability that it provides, it undergoes rapid hydrolysis by carboxylesterase 1 in the liver to form an inactive metabolite, CNS 7054 [4]. Therefore, unlike midazolam, remimazolam exhibits a swift and predictable onset and offset due to its small volume of distribution and high clearance [5]. Additionally, the use of flumazenil, a specific antagonist, enables rapid recovery from remimazolam-induced anesthesia. These attributes make remimazolam particularly valuable for the short-term sedation of patients undergoing procedures or daytime surgeries that necessitate short periods of sedation and rapid recovery.

Remimazolam is used primarily for its sedative properties, while opioids, such as remifentanil, are concurrently administered to manage pain control [6]. In our previous research, we identified the median effective dose of remimazolam when combined with remifentanil for the facilitation of i-gel supraglottic airway (ISA) insertion in adult patients [7]. As evidence suggesting significant physiological and pharmacological variations between men and women in response to anesthetic agents are emerging, we focused on the sex differences in the requirement of remimazolam for ISA insertion [8,9]. This can impact drug efficacy and safety, necessitating a tailored approach to dosage. In anesthetic practice, recognizing and accommodating sex-based differences is crucial for optimizing patient outcomes and enhancing the safety and effectiveness of procedures like ISA insertion. This study, therefore, aimed to investigate the 50% and 95% effective bolus doses (ED_50_ and ED_95_) of remimazolam for ISA insertion under remifentanil infusion in both men and women.

## 2. Materials and Methods

This prospective study was approved by the Institutional Review Board of the Ajou University Hospital (Suwon, South Korea; AJOUIRB-IV-2022-440) and registered with the Clinical Research Information Service (CRIS No. KCT0007906, registration date: 17 November 2022). After obtaining written informed consent from all patients, the study was conducted in accordance with the 2013 Declaration of Helsinki. Patients were enrolled from December 2022 to August 2023 and were aged 19–65 years, classified according to the American Society of Anesthesiologists physical status as class 1 or 2, scheduled for general anesthesia, and capable of undergoing ISA insertion. Those who met any of the following criteria were excluded from the study: a body mass index >30 kg/m^2^; expected difficulties with airway management or mask ventilation; a history of any chronic obstructive pulmonary disease, asthma, or pneumonia; an active upper respiratory infection; an elevated risk of aspiration; previous allergic reactions to benzodiazepines or opioids; a history of habitual use of benzodiazepines or opioids; substantial kidney or liver impairment (estimated glomerular filtration rate < 30 mL/min/1.73 m^2^, Child–Pugh score ≥ 7); and being pregnant or breastfeeding. Patients were divided into two groups based on their sex.

When the patient arrived in the operating room, standard monitoring was initiated, including electrocardiography, noninvasive blood pressure measurement, peripheral pulse oximetry, and bispectral index (BIS) monitoring (A-2000; Aspect Medical Systems, Newton, MA, USA). For anesthesia induction, effect-site target-controlled infusion (TCI) was initiated using an infusion device (Orchestra Base Primea; Fresenius Vial, Brézins, France), with a remifentanil effect-site concentration (Ce) set at 3.0 ng/mL [10]. One researcher (J.O) prepared and pre-calculated the remimazolam (Byfavo; Hana Pharm Co., Ltd., Seoul, Republic of Korea) dose (1 mg/mL in a 50-mL syringe). Once the Ce of remifentanil reached 3.0 ng/mL, the pre-calculated dose of remimazolam was administered. Another researcher (S.Y.P), who was blinded to the remimazolam dose, evaluated the patient’s loss of consciousness (LOC) and inserted the ISA. LOC was assessed for 150 s after administration of the remimazolam bolus [6,7,11] by using the Modified Observer’s Assessment of Alertness/Sedation (MOAA/S) score [12]. The ISA was inserted when the following conditions were met: (1) the MOAA/S score was 0 or 1, (2) the response to jaw thrusting was lost [13], and (3) the Ce of remifentanil was 3.0 ng/mL. For the patient’s safety, ISA insertion was attempted only once. Optimal ISA insertion was defined as adequate ventilation (a rectangular capnography waveform on the ventilator) after placement of the ISA without body movement, coughing, or gagging. For patients not exhibiting LOC or cases of unsuccessful ISA insertion, we promptly initiated inhalation of 2.0–2.5% sevoflurane and concurrently administered 0.6 mg/kg of body weight of rocuronium for their safety. Once the ISA was successfully inserted, we immediately stopped remifentanil infusion and started remimazolam infusion at 1–2 mg/kg/h to maintain sedation, as recommended by the manufacturer. During the procedure, we recorded the patient’s vital signs and BIS values at various time points: before anesthesia induction (T_0_), at the point of LOC (T_1_), immediately after ISA insertion (T_2_), 1 min after insertion (T_3_), 3 min after insertion (T_4_), 5 min after insertion (T_5_), and 10 min after insertion (T_6_). Measurements from T_2_ to T_6_ were collected only for patients with both LOC and successful ISA insertion. During the study period, hypotension was defined as mean arterial blood pressure < 65 mmHg or systolic arterial blood pressure (SBP) decreased by >30% from the baseline [14]. When hypotension occurred, 4–8 mg of ephedrine was intravenously administered. Additionally, if the patient’s heart rate (HR) dropped below 45 bpm for more than 1 min, we intravenously administered 0.5 mg of atropine.

Patients were sequentially enrolled using the modified version of Dixon’s up-and-down method [15,16]. The starting dose of remimazolam in both sex groups was set at 0.25 mg/kg, based on our previous study results [7], with a step size of 0.05 mg/kg. The dose was adjusted up or down depending on whether the previous patient responded negatively or positively to the drug. That is, when ISA insertion was successful, the dose was decreased by 0.05 mg/kg for the next patient; conversely, if insertion failed, the remimazolam dose was increased by 0.05 mg/kg for the next patient. After the patients were transferred to the post-anesthesia care unit, postoperative nausea and vomiting, sore throat, and memory recall were assessed.

### Statistical Analyses

The primary outcomes of this study were the ED_50_ and ED_95_ of remimazolam required to achieve an adequate depth of anesthesia for ISA insertion. To obtain the ED_50_ via the modified Dixon’s up-and-down method, a minimum of six crossover pairs in the same direction, approximately 10 changes in direction, and at least 20 patients were required [17]. In this study, the sequence was continued until seven crossover pairs were attained for each group to satisfy the conditions. The ED_50_ and ED_95_ values of remimazolam were determined using isotonic regression with a pool-adjacent-violators algorithm (PAVA) along with confidence intervals (CIs) [18]. When the 83% and 95% CIs of the ED_50_ and ED_95_ values, respectively, did not overlap, the values were considered significantly different [19]. 

IBM SPSS Statistics for Windows (version 25.0; IBM Corporation, Armonk, NY, USA) and R software (version 4.0.5; R Foundation for Statistical Computing, Vienna, Austria) was used to perform statistical analyses. Categorical variables were assessed using the chi-squared test and presented as frequencies. Continuous variables were evaluated using the Student’s *t*-test or Mann–Whitney test and expressed as means ± standard deviations or as medians with the interquartile range (25th to 75th quartile). The normality of the continuous data was assessed using the Shapiro–Wilk test. Repeated measurements of variables were subjected to one-way repeated-measures analysis of variance. Where the model indicated a significant interaction between sex group and time, a post hoc analysis was conducted to identify the time points with notable differences. Variables were considered statistically significant when the *p*-value was less than 0.05.

## 3. Results

**Figure 1:**
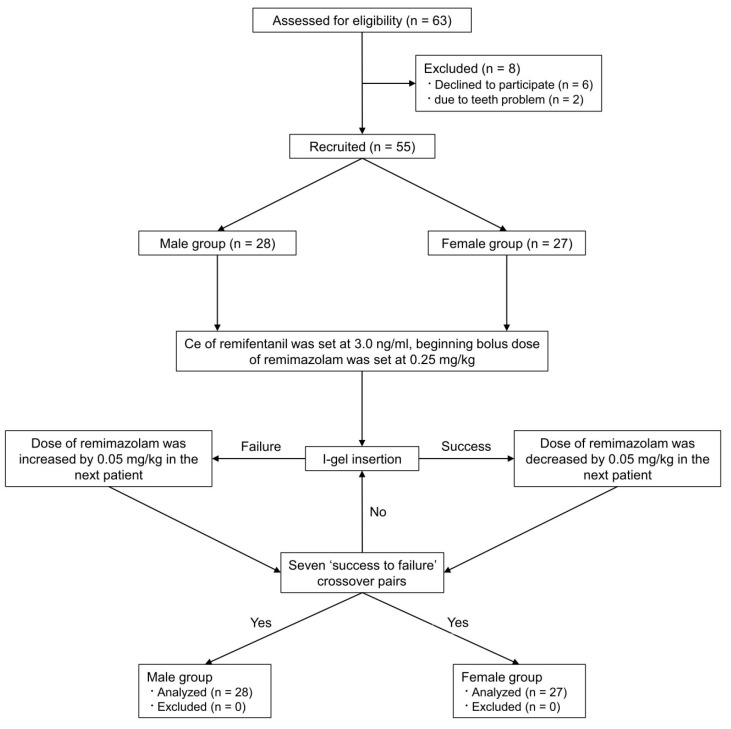
Flow diagram for this study.

Among the 63 patients who were screened for eligibility, two withdrew owing to tooth problems, and six were excluded owing to a lack of consent for participation in the study. Finally, 55 patients (28 men and 27 women) were enrolled and analyzed (Figure 1).

**Figure 2:**
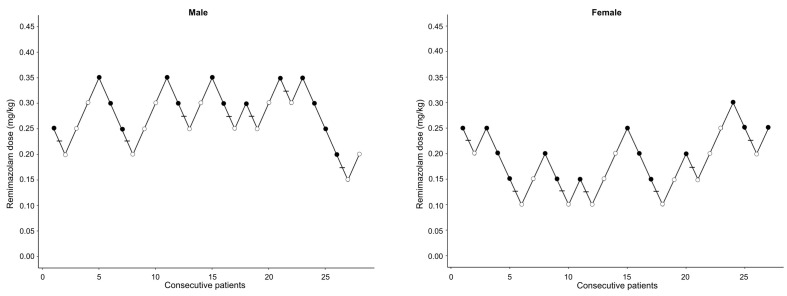
The responses of consecutive male and female patients to i-gel supraglottic airway insertion. Successful insertions are denoted by solid circles; failed insertions are denoted by open circles. Horizontal bars represent crossover midpoints (success-to-failure).

**Figure 3:**
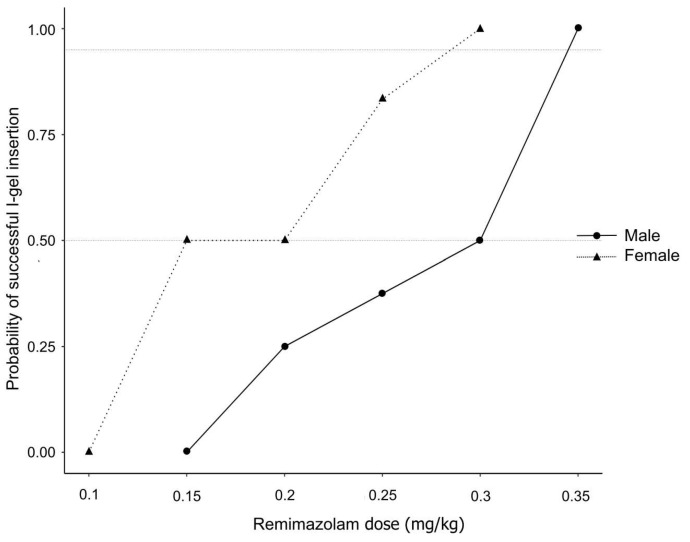
The pool-adjacent-violators algorithm probability of successful i-gel supraglottic airway insertion according to the remimazolam dose co-administered with remifentanil at an effect-site concentration of 3 ng/mL.

Table 1 presents the patients’ demographic characteristics. Figure 2 illustrates the remimazolam dose according to the outcome of ISA insertion for each consecutive patient. The ED_50_ of remimazolam (mean ± standard error), calculated via Dixon’s up-and-down method, was 0.28 ± 0.02 mg/kg in men and 0.18 ± 0.02 mg/kg in women (*p* < 0.001). According to the isotonic regression analysis, the ED_50_ of remimazolam (83% CI) was 0.30 (0.27–0.32) mg/kg in the male group and 0.20 (0.15–0.23) mg/kg in the female group. The ED_95_ (95% CI) in the male group was 0.35 (0.34–0.35) mg/kg and 0.29 (0.25–0.30) mg/kg in the female group (Table 2). Figure 3 depicts the isotonic regression analysis using PAVA and the bootstrapping approach.

We compared vital signs and BIS changes at each data collection point from their respective baseline measurements (Table 3). A notable decrease in blood pressure was observed compared to the baseline measurement. Three men and five women had hypotension (SBP dropped >30% from baseline) that necessitated ephedrine injection. Blood pressures exhibited similar tendencies over time in the two groups, with no significant differences between the male and female groups. However, the HR significantly differed between the male and female groups from T_2_ to T_6_. During this period, the HR of the female group was significantly lower than that of the male group. Additionally, the male group had significantly lower BIS values than did the female group from T_4_ to T_6_. No patients experienced memory recall, even though some had a BIS index higher than 60 (men: 7/28, 25.0%; women: 12/27, 44.4%).

## 4. Discussion

The differences between the sexes concerning the way anesthetics are processed and their pharmacokinetic (PK) and pharmacodynamic (PD) effects are gaining scientific interest. To date, no reports on disparities between the sexes have been published regarding the amount of remimazolam required for supraglottic airway insertion in non-paralyzed patients when combined with remifentanil. However, our study revealed that, during remifentanil TCI, men required significantly higher doses of remimazolam (both ED_50_ and ED_95_) for successful ISA insertion than women. This suggests that, while administering remimazolam for ISA insertion, the sex of the patient should be considered for appropriate dosing.

For the insertion and maintenance of a supraglottic airway device, the airway reflex must be suppressed, especially the cough reflex. The cough reflex is a defensive reflex for the clearance of secretions and aspirated particles to protect the airways and lungs [20]. The non-myelinated vagal afferent C-fibers and myelinated cough receptors, which innervate the mucosa of the larynx, trachea, carina, and bronchi, are sensitive to chemical and mechanical stimuli and interact with the brainstem to regulate coughing [21]. Several antitussive agents, such as opioids and local anesthetics, inhibit these cough pathways [22]. The anesthetic concentration for cough suppression may differ between the sexes in similar clinical settings. As women have higher sensitivity to μ-opioid receptor agonists and higher binding affinity to μ-opioid receptors than do males, the antitussive effect of opioids is more potent in women than in men [23,24]. Therefore, in this study, men could require a larger dose of remimazolam to suppress the airway response compared to women. However, as we used the Minto model for remifentanil administration, which is known to not yield sex-related differences in PKs or PDs [25], we believe that the impact of remifentanil on the required remimazolam dosage for ISA placement would be relatively small. Therefore, the main cause of the sex disparity in the dose of remimazolam to inhibit the airway reflex may be caused by the pharmacological aspects of remimazolam. As benzodiazepines have minimal antitussive effects, a sufficient sedation depth should be required to avoid adverse upper airway responses evoked by ISA insertion.

The potential impact of sex differences on PKs depends on the drug distribution, metabolism, and clearance [26]. Women have a higher body fat percentage and lower water content than men, which may affect the drug distribution. Typically, for lipophilic drugs, such as opioids and benzodiazepines, the drug distribution volume per body weight tends to be higher in women. Thus, administering the same dose per body weight will lead to a lower central drug distribution volume of lipophilic drugs in women than in men, which means women will require a larger bolus dose for the same effect than men. As the central drug distribution volume is a crucial PK factor that influences the initial drug concentration after a single-dose administration, these sex differences affect the ideal dose. However, a study by Zhou et al. found no sex difference on the central drug distribution volume of remimazolam [27]. Consequently, the sex-related dose difference in our study is likely attributed to differences in the PDs. According to Wiltshire et al. [28], PD models using BIS monitoring revealed that men are twice as sensitive to remimazolam compared to women, which contradicts our results. However, Wiltshire et al. [28] compared only the decrease in BIS, whereas we compared the successfulness of deep sedation for inserting the ISA. Hence, sex-related sensitivity to remimazolam may vary depending on the primary outcome of interest. In a previous study, the remimazolam dosage for supraglottic airway insertion, when combined with sufentanil, was also significantly higher in men than in women, which is consistent with our results [29].

The influence of the menstrual cycle on anesthetic drugs is an evolving area of research that warrants further exploration [30,31]. A previous study by Fu et al. [32] noted that menstrual cycle phases influenced the 50% effective effect-concentration of propofol for LOC, with lower requirements in the luteal phase compared to the follicular phase due to progesterone variations. It is conceivable that similar hormonal effects could be influencing the pharmacodynamics of remimazolam. Our findings, which indicate a sex difference in remimazolam requirements for I-gel insertion, may therefore be partly attributed to menstrual cycle-related hormonal variations, mirroring the sex-specific responses observed in propofol studies.

This study revealed significant differences in the HR and BIS value at certain time points between the two groups. In the female group, the HR was significantly lower than that in the male group from the moment the ISA was inserted, which continued up to 10 min after the insertion. In men, the BIS value was lower 3–10 min after the ISA was inserted than that in women. This observation is likely associated with the higher dosage requirement for remimazolam in the male group, as the plasma concentrations of hypnotic agents, such as propofol, midazolam, and volatile agents, are significantly correlated with BIS values [33,34,35]. Also, according to Wiltshire et al., the sensitiveness of BIS decrement is twice as sensitive in men compared to women. Whether HR temporarily increases owing to remimazolam remains unclear because of inconsistencies across studies and observed time points. Certain reports have revealed the incidence of bradycardia when remimazolam was used for general anesthesia or procedural sedation [36,37]. However, in a PK-PD study of remimazolam, the HR increased by 28 ± 15% [11]. Resolving this inconsistency would require further research on the relationship between HR and remimazolam. Our sample size was estimated using Dixon’s up-and-down allocation approach and might have been insufficient to confirm differences in secondary outcomes between the two groups.

This study has several limitations. First, remifentanil, which was co-administered with remimazolam, may exhibit different analgesic and antitussive potencies between men and women. We cannot exclude the influence of remifentanil on the remimazolam dose required for the insertion of the ISA. This might have occurred despite our efforts to minimize sex differences by employing the Minto model. Second, the ED_95_, calculated using an up-and-down sequential allocation design with a focus on the ED_50_, may not be a reliable measure. Further studies using methods such as the biased coin up-and-down method for a more accurate evaluation of the ED_95_ for remimazolam may assist in establishing appropriate dosage requirements for the different sexes. Third, although the sequential method is indeed an efficient and convenient approach for swift determination of the effective dose in a small sample, the limited sample size constitutes a weakness of this study. Fourth, in instances where the BIS exceeded 60 but was not correlated with raw electroencephalogram data, we acknowledge the potential for misleading interpretations regarding patient consciousness [38]. Considering other studies, there seems to be a tendency for higher BIS values when using remimazolam without any incidences of awareness with recall, highlighting the necessity for a cautious interpretation of BIS values when using remimazolam [39]. Lastly, we did not specifically investigate the potential influence of the menstrual cycle in female patients. This consideration is indeed pertinent, as hormonal fluctuations during the menstrual cycle can significantly impact drug metabolism and response. Future studies are warranted to explore this aspect in greater detail, which could yield valuable insights into sex-specific remimazolam responses.

In conclusion, when remimazolam was used in combination with remifentanil, men required higher ED_50_ and ED_95_ values to facilitate ISA insertion as compared to women. Therefore, while using remimazolam alongside remifentanil infusion without NMBAs for ISA insertion, one should consider the patient’s sex for appropriate dosing.

## Figures and Tables

**Table 1:** Demographic characteristics of patients.

Parameters	Men (n = 28)	Women (n = 27)	*p* Value
Age (years)	41.5 (29.5, 49.0)	50.0 (35.5, 54.0)	0.206
Weight (kg)	77.2 ± 9.6	61.5 ± 6.9	*<0.001*
Height (cm)	173.9 ± 6.9	159.1 ± 5.5	*<0.001*
BMI (kg/m^2^)	25.9 (24.0, 27.1)	24.6 (22.3, 26.1)	0.072
ASA PS class			0.701
I	21 (75.0)	18 (66.7)	
II	7 (25.0)	9 (33.3)	

Values are presented as mean ± standard deviation, median with the interquartile range (25th to 75th quartile), or number of patients (percentage). BMI, body mass index; ASA PS, American Society of Anesthesiologists physical status. *Italics* indicate *p* Values less than 0.05.

**Table 2:** Dose of remimazolam required for the smooth insertion of i-gel supraglottic airway.

Parameters	Men (n = 28)	Women (n = 27)	*p* Value
Modified Dixon’s up-and-down method			
ED_50_, mg/kg	0.28 ± 0.02	0.18 ± 0.02	*<0.001*
Isotonic regression method			
ED_50_ (83% CI), mg/kg	0.30 (0.27–0.32) *	0.20 (0.15–0.23)	
ED_95_ (95% CI), mg/kg	0.35 (0.34–0.35) *	0.29 (0.25–0.30)	

The ED_50_ calculated by modified Dixon’s up-and down method is presented as the mean ± standard error. The ED_50_ (83% CI) and ED_95_ (95% CI) were determined using the isotonic regression method. * Values significantly higher in men than those in women (non-overlapping CI method). ED_50_, effective dose in 50% of the sample; ED_95_, effective dose in 95% of the sample; CI, confidence interval. *Italics* indicate *p* Values less than 0.05.

**Table 3:** Dose of changes in mean arterial pressure, heart rate, and bispectral index during the study period.

Time	Men (n = 14)	Women (n = 14)	*p* Value
Mean arterial pressure			
T_0_	101.00 ± 11.86	108.07 ± 17.80	0.227
T_1_	90.43 ± 15.85 *	88.79 ± 13.27 *	0.769
T_2_	85.50 ± 12.79 *	82.21 ± 14.12 *	0.524
T_3_	83.14 ± 12.11 *	79.71 ± 12.65 *	0.470
T_4_	80.64 ± 12.04 *	79.36 ± 11.56 *	0.775
T_5_	85.07 ± 12.90 *	82.29 ± 13.60 *	0.583
T_6_	86.93 ± 15.15 *	88.00 ± 15.70 *	0.856
Heart rate			
T_0_	68.93 ± 13.20	68.64 ± 10.95	0.951
T_1_	75.14 ± 14.39 *	68.79 ± 9.24	0.176
T_2_	73.43 ± 10.39	63.43 ± 9.40 *	*0.013*
T_3_	72.64 ± 10.72	62.29 ± 9.86 *	*0.013*
T_4_	75.50 ± 11.59 *	63.36 ± 10.80 *	*0.008*
T_5_	78.07 ± 10.09 *	67.50 ± 9.04	*0.007*
T_6_	77.57 ± 9.58 *	68.79 ± 10.21	*0.027*
Bispectral index			
T_0_	96.86 ± 1.29	94.14 ± 3.46	*0.011*
T_1_	61.79 ± 9.46 *	61.36 ± 12.29 *	0.918
T_2_	60.64 ± 6.42 *	64.07 ± 10.58 *	0.309
T_3_	58.50 ± 5.60 *	63.21 ± 8.61 *	0.098
T_4_	56.86 ± 4.66 *	64.50 ± 8.77 *	*0.008*
T_5_	58.36 ± 4.65 *	65.57 ± 7.67 *	*0.006*
T_6_	56.71 ± 5.54 *	61.86 ± 7.85 *	0.056

Values are presented as mean ± standard deviation. T_0_, baseline; T_1_, loss of consciousness; T_2_, immediately after i-gel supraglottic airway insertion; T_3_, 1 min after insertion; T_4_, 3 min after insertion; T_5_, 5 min after insertion; T_6_, 10 min after insertion. * *p* < 0.05 compared to baseline within the group. *Italics* indicate p Values less than 0.05.

## Data Availability

The data presented in this study are available on request from the corresponding author.
